# Do users of long-acting reversible contraceptives receive the same counseling content as other modern method users? A cross-sectional, multi-country analysis of women's experiences with the Method Information Index in six sub-Saharan African countries

**DOI:** 10.1016/j.conx.2022.100088

**Published:** 2022-11-11

**Authors:** Brooke W. Bullington, Katherine Tumlinson, Celia Karp, Leigh Senderowicz, Linnea Zimmerman, Pierre Z. Akilimali, Musa Sani Zakirai, Funmilola M. OlaOlorun, Simon P.S. Kibira, Frederick Edward Makumbi, Solomon Shiferaw, PMA Principal Investigators Group

**Affiliations:** a Department of Epidemiology, Gillings School of Global Public Health, University of North Carolina at Chapell Hill, Chapel Hill United States of America; b Carolina Population Center, University of North Carolina at Chapel Hill, Chapel Hill United States of America; c Department of Maternal and Child Health, Gillings School of Global Public Health, University of North Carolina at Chapell Hill, Chapel Hill United States; d Department of Population, Family and Reproductive Health, Johns Hopkins Bloomberg School of Public Health, Baltimore United States; e Departments of Gender and Women's Studies and Obstetrics and Gynecology, University of Wisconsin-Madison, Madison United States; f Kinshasa School of Public Health, University of Kinshasa, Kinshasa, Democratic Republic of the Congo; g National Population Commission, Abuja, Nigeria; h College of Medicine, University of Ibadan, Nigeria; i Department of Community Health and Behavioural Sciences, School of Public Health, Makerere University, Kimpala, Uganda; j Department of Epidemiology and Biostatistics, School of Public Health, Makerere University, Kimpala, Uganda; k Department of Reproductive and Health Services Management, Addis Ababa University, Ethiopia

**Keywords:** Contraception, Family planning, Informed choice, Long-acting reversible contraception

## Abstract

**Objective:**

There has been a growing focus on informed choice in contraceptive research. Because removal of long-acting reversible contraception (LARC), including implants and IUDs, requires a trained provider, ensuring informed choice in the adoption of these methods is imperative. We sought to understand whether information received during contraceptive counseling differed among women using LARC and those using other modern methods of contraception.

**Study Design:**

We used cross-sectional data from Burkina Faso, Côte d'Ivoire, the Democratic Republic of Congo (DRC), Kenya, Nigeria, and Uganda collected in 2019–2020 by the Performance Monitoring for Action project. We included 7969 reproductive-aged women who reported use of modern contraception. Our outcome of interest, information received during contraceptive counseling, was measured using a binary indicator of whether respondents answered “yes” to all 4 questions that make up the Method Information Index Plus (MII+). We used modified Poisson models to estimate the prevalence ratio between method type (LARC vs. other modern methods) and the MII+, controlling for individual- and facility-level covariates.

**Results:**

Reported receipt of the full MII+ during contraceptive counseling ranged from 21% in the DRC to 51% in Kenya. In all countries, a higher proportion of LARC users received the MII+ compared to other modern method users. A greater proportion of LARC users answered “yes” to all questions that make up the MII+ at the time of counseling compared to other modern method users in DRC, Kenya, Nigeria, and Uganda. There was no significant difference in the prevalence of reporting the full MII+ between users of LARC and other modern methods in Burkina Faso (Adjusted prevalence ratio (aPR): 1.16; 95% confidence interval (CI): 0.91, 1.48) and Côte d'Ivoire (aPR: 1.13; 95% CI: 0.87, 1.45).

**Conclusion:**

Information received during contraceptive counseling was limited for all modern contraceptive users. LARC users had significantly higher prevalence of receiving the MII+ compared to other modern method users in the DRC, Kenya, and Uganda. Family planning programs should ensure that all women receive complete, unbiased contraceptive counseling.

**Implications:**

Across 6 sub-Saharan African countries, a substantial proportion reproductive-aged women using contraception did not report receiving comprehensive counseling when they received their method. Women using long-acting reversible contraception received more information compared to women using other modern methods in the DRC, Kenya, Nigeria, and Uganda after controlling for individual- and facility-level factors.

## 1 Introduction

Quality of care is an important component of contraceptive services. Judith Bruce's seminal 1990 framework for conceptualizing quality emphasized “information given clients” as a key element of high-quality contraceptive care and highlighted the importance of quality for promoting person-centered family planning programming [1]. In the years since, there has been a growing focus on the concept and measurement of informed choice, which explores whether an individual has sufficient, unbiased information about a range of options when making a decision about contraception [2], [3], [4]. Ensuring the protection of human rights in reproductive services, including contraceptive autonomy, requires concerted and consistent focus on the measurement of informed choice.

Measuring informed choice and quality of contraceptive care among users of long-acting reversible contraception (LARC), specifically contraceptive implants and intrauterine devices (IUDs), is vital given that removal requires a trained provider. Users are therefore dependent on the training, availability, and willingness of providers to successfully discontinue their method. While LARC is highly-effective and generally has high rates of satisfaction [5], [6], [7], [8], the reliance on providers has raised concerns about potential threats to autonomy, particularly if women are unable to discontinue on demand [9], [10], [11]. Researchers found that providers imposed method restrictions in Nigeria, Kenya, Senegal, and India based on age, parity, and marital status, with stringent restrictions preventing women from accessing IUDs [12], [13], [14], [15], [16], [17]. Similarly, studies in Kenya, Ethiopia, and Ghana have reported provider refusal to remove LARC upon the client's request [18], [19], [20]. In order for women to make informed decisions that balance their preferences across a range of factors, including user engagement, efficacy, potential for side-effects, and reliance on providers, it is imperative that those using LARC receive comprehensive counseling centered on informed choice.

One of the ways the family planning community has measured informed choice as an integral element of quality care is through the Method Information Index (MII). The MII captures three components of a counseling visit, relating to counseling on other methods, side effects, and what to do in case of side effects [21]. In 2019, the MII was adapted into the MII+ with the addition of a fourth question about whether an individual was told about the possibility of switching to another method [22].

Despite its wide adoption by researchers and policy makers to evaluate the success of family planning programs, including FP2020 [23], some scholars have questioned the validity of the measure, noting that it may overestimate the information exchanged between patients and providers during counseling [24]. Additionally, while the MII was designed to measure information given to clients during counseling, it has been routinely adopted as a proxy for the full spectrum of informed choice or for the construct of quality of care writ large [25], [26], [27], [28]. This may be problematic given that the MII does not include items to capture information on correct method use, warning signs, or the range of methods presented to the client, among other elements important to informed decision-making. To date, however, the MII+ remains the only population-based measure of contraceptive quality widely measured in large-scale demographic surveys, such as the Demographic and Health Surveys.

Previous attempts to measure informed choice for LARC have found that quality and content of contraceptive counseling may differ between LARC users and users of other modern methods. Studies focused on predictors of the MII have reported that women using LARC were more likely to report receiving all components of the MII compared to users of other modern methods and general recipients of contraceptive counseling [26,[29], [30], [31]]. Additionally, a qualitative study in Tanzania reported that women received biased contraceptive counseling following a provider-focused postpartum IUD intervention, with the IUD promoted over other methods [32]. Though previous studies have documented contraceptive counseling differences between LARC and non-LARC users, no quantitative study has explored the association between method and MII+ across multiple countries, adjusting for relevant confounders of this relationship. By conducting this analysis across a number of sub-Saharan countries, we are better able to articulate the differences in the provision of MII+ components during contraceptive counseling in this region of the Global South.

The aim of the present study is to understand if and how reported receipt of the MII+ at the time of contraceptive counseling differs between women using LARC (implants and IUDs) and those using other modern methods of contraception (pills, injectables, female condom, sterilization, emergency contraception, lactational amenorrhea method, and the Standard Days Method). We use nationally representative, population-based data from six sub-Saharan African countries to examine the association between modern method used and reported receipt of all four components that make up the MII+. We explore variability of the MII+ across a range of geoculturally diverse contexts and assess differences in the MII+ between LARC and non-LARC users.

## 2 Methods

### 2.1 Data and study setting

We use data collected in 2019–2020 by the Performance Monitoring for Action (PMA) project, which conducts nationally and regionally representative surveys of women aged 15 to 49 in sub-Saharan Africa and South Asia. Participants were selected using a multistage sampling strategy. First, a representative sample of enumeration areas (EAs) were selected. Households were then selected at random from a list of all households in each EA. All reproductive-aged women residing in randomly selected households were invited to participate in a female questionnaire after providing informed consent. Eligible women ages 15 to 49 provided oral or written consent to participate, providing information about their sociodemographic characteristics, fertility preferences, reproductive and contraceptive behaviors, and other related information. Additional detail about the sampling strategy and survey procedures can be found in Zimmerman et al. and www.pmadata.org [33].

Study geographies include Burkina Faso, Côte d'Ivoire, Democratic Republic of Congo (DRC, Kinshasa and Kongo Central), Kenya, Nigeria (Kano and Lagos states), and Uganda. In these countries, modern contraceptive use ranges from 18% in Nigeria to 25% in Kenya and Burkina Faso. LARC use among family planning users ranges from 33% in Côte d'Ivoire to 58% in Burkina Faso. LARC use in these geographies is dominated by the implant, which ranges from 29% of the method mix in Côte d'Ivoire to 53% in Burkina Faso. IUD use ranges from 4% in Kenya and Côte d'Ivoire to 10% in Nigeria.

### 2.2 Measures

Our primary outcome is information shared during contraceptive counseling measured by the MII+. Current and recent family planning users were asked to reflect back on their last counseling visit and asked, “when you obtained your [current or most recent method], were you told about:” 1) side effects or problems you might have with your method?, 2) what to do if you experienced side effects? 3) other methods that you could use?, and 4) the possibility of switching to another method if the method you selected was not suitable? For each question, participants responded yes/no. Each individual component of the MII+ is treated as a binary variable (yes/no). We use a binary indicator (yes/no) of “reported receipt of the complete MII+” for those who responded “yes” to all 4 counseling components [22,28]. Our primary exposure is LARC use, defined as either use of a LARC method (implant or IUD) or another modern method (pills, injectables, female condom, sterilization, emergency contraception, lactational amenorrhea method, and the Standard Days Method).

Our analysis includes a number of covariates at the individual level, which we both describe and include in our models. We identified these covariates using a directed acyclic graph. Covariates include age (continuous), marital status (married/unmarried), and parity (continuous). Information on the facility where the woman received her contraceptive method was self-reported, including facility ownership (public facility vs. other), type (hospital vs. other facility type), and urbanicity of clientele (urban/rural).

### 2.3 Sample

We restrict our analytic sample to modern contraceptive method users. We exclude women who reported male condom use as their only form of contraception, as it is common for men to obtain condoms themselves, precluding many women from contraceptive counseling. We also exclude the small number of women for whom data on all four aspects of the MII+ were not collected due to non-response (Burkina Faso N = 6; Côte d'Ivoire N = 12; Democratic Republic of Congo N = 51; Kenya N = 80; Nigeria N = 17; Uganda N = 35).

### 2.4 Analysis

We describe sociodemographic characteristics of modern contraceptive users by country and present the proportion of women who received each of the components of the MII+ and all four components, stratified by LARC use status (LARC users vs. other modern method users). Finally, we use modified poisson regression with a log link to estimate prevalence ratios for the association between a binary indicator of women's method type (LARC vs. other modern method) and reported receipt of the complete MII+ (yes/no), adjusting for age, marital status, parity, urbanicity, and facility ownership and type where the woman sourced her contraceptive method. All analyses were weighted to account for the complex sampling design and stratified by country to explore contextual variations in this relationship.

## 3 Results

Altogether, 7969 modern contraceptive users were included across the six geographies. Of women included, LARC users accounted for 58% of women in Burkina Faso, 33% in Côte d'Ivoire, 46% in DRC, 46% in Kenya, 43% in Nigeria, and 42% in Uganda. The implant was the most common modern method of contraception in Burkina Faso (53%), Côte d'Ivoire (29%), the Democratic Republic of Congo (44%), Kenya (42%), and Nigeria (33%), followed by the injectable. In contrast, in Uganda, the injectable was the most common modern contraceptive method (41%), followed by the implant (36%). Across all countries, most women were married (67–91%). In our sample, between 30 and 40% of women had 1 to 2 children, 26 to 43% of women had 3 to 4 children, and 24 to 38% of women had 5 or more children.

Across geographies, receipt of counseling content varied widely. Roughly 47 to 69% of women were told about side effects of their method at the time of contraceptive counseling, 37 to 63% of all women were told what to do if they experienced side effects, 40 to 74% were told about other methods, and 43 to 71% were told they could switch to another method. Thus, in all countries, fewer than 80% of participants reported receipt each of the individual components that make up the MII+ indicator. Receiving information about method switching during counseling was the most frequently reported component of the MII+ in Burkina Faso (71%), Côte d'Ivoire (55%), Kenya (77%), and Uganda (70%). In the DRC, receiving information about side effects was the most frequently reported component of the MII+ (55%) and in Nigeria, receiving information about other contraceptive methods was the most frequently reported component (74%) of the MII+.

**Fig. 1:**
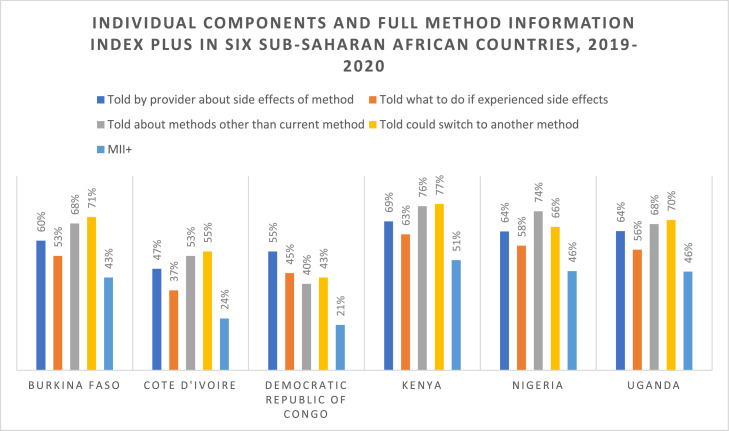
Individual components and full Method Information Index Plus in six sub-Saharan African countries, 2019-2020.

Reported receipt of all 4 components of the MII+ was low across all countries, ranging from 21 to 51%. Kenya had the highest proportion of women who reported receipt of the full MII+ (51%), followed by Nigeria (46%), Uganda (46%), Burkina Faso (43%), Côte d'Ivoire (24%), and the DRC (21%). Individual components and the full MII+ are shown by country in Figure 1.

**Fig. 2:**
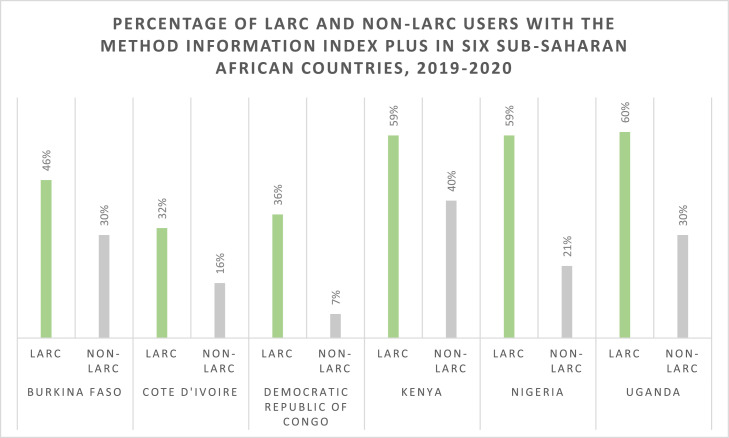
Percentage of long-acting reversible contraception (LARC) users and non-LARC users with the Method Information Index Plus in six sub-Saharan African countries, 2019-2020.

Among LARC users, 32 to 60% reported receipt of the full MII+, compared to 8 to 45% of users of other modern methods. In all countries, a higher proportion of LARC users reported receipt of each individual component of the MII+ and the full MII+ compared to other modern method users (Table 2, Fig. 2). The difference in reported receipt of MII+ comparing LARC users and other modern method users was smallest in Burkina Faso (46% of LARC users vs. 40% of non-LARC users) and largest in the DRC (36% of LARC users vs. 8% of non-LARC users). In Burkina Faso, the DRC, Kenya, and Uganda, the proportion of women told what to do in case of side effects was the element of the MII+ with the largest difference between LARC and non-LARC users.

Crude and adjusted prevalence ratios estimating the relationship between LARC use and reported receipt of all 4 MII+ components, stratified by country, are presented in Table 3. The prevalence of the MII+ was significantly higher among those using LARC compared to those using a other modern methods in the DRC, Kenya, Nigeria, and Uganda. In Burkina Faso and Côte d'Ivoire, there were no significant differences in reported receipt of the MII+ between LARC users and other modern method users. Adjusted prevalence ratios ranged from 1.13 in Côte d'Ivoire (95% CI: 0.87 1.45) to 3.65 in the DRC (95% CI: 1.52, 8.75).

## 4 Discussion

In this multicountry analysis, we find that information received during contraceptive counseling, an important aspect of informed choice, was limited for all modern contraceptive users with considerable differences between LARC users and other modern method users. Across 6 countries, fewer than 60% of women reported receiving counseling on side effects, what to do in case of side effects, other contraceptive methods, and method switching. Contrary to our hypothesis, women using LARC had significantly higher prevalence of reporting the full MII+ at the time of counseling compared to women using other modern methods in the Democratic Republic of Congo, Kenya, Nigeria, and Uganda.

Reported receipt of all components of the MII+ differed substantially by country, ranging from 51% in Kenya to 21% in the DRC. These findings are in line with studies that have previously examined the MII in the included countries and reflect the range of information given to contraceptive clients across sub-Saharan African countries [31,34, 35, 22]. Previous studies have found that clients who did not receive counseling on all components of the MII are more likely to discontinue their method within a year of initiating [26,36]. Discontinuation may be an example of women exercising autonomy over their contraception use, and it may also reflect insufficient counseling on method management prior to adoption. Improving counseling given to clients is therefore imperative to ensure that all people have the information they need before choosing which method to adopt.

Our study found similar associations to previous studies that examined LARC use as a predictor of the MII, and built on this work by delving into the individual components of the MII+ [26,29]. Similar to studies in Kenya, Togo, and Ethiopia, we find that women using LARC were more likely to have the full MII+ compared to women using other modern methods [26,30,34]. We report that a higher proportion of LARC users received each component of the MII+ compared to users of other modern methods in all countries. This suggests that our findings were not driven by one component of informational counseling, but rather that LARC users were overall provided more information during counseling sessions. This may be because LARC requires longer interactions with providers, and thus clients have more time to be exposed to information about their method and ask questions. It may also be that providers delivering LARC have more experience than those delivering other methods and are therefore more confident in counseling. The largest driver of difference in the MII+ between LARC and non-LARC users was either being told about side effects or what to do in case of side effects in 5 of the 6 countries. Results from this analysis suggest that women who reported using LARC seem to be at a advantage in receipt of counseling components as measured by the MII+, even after controlling for factors, like facility-type, that likely influence receipt of the MII+.

Previous research has highlighted limitations to the MII measure that have important implications for these results. In 2019, Chang et al. [24] assessed the MII by comparing the binary questions that make up the measure to detailed questions about what information was shared during counseling, asking participants to list what other methods they knew, the side effects of their method, and what specifically they were told to do in case of side effects. They found that adjusting the MII for discordance between the MII score and actual knowledge after counseling led to significant reductions in the indicator. These reductions were largest among users of the IUD and considerable among implant users, indicating that the validity of the MII may be different for LARC users and users of other methods. Though we report that women using LARC had higher prevalence of receiving the MII+, differential measurement of our outcome (the MII+) by our exposure (method type) may bias these findings. If this is the case, estimates presented in this manuscript may overestimate the true association between method type and MII. Further research exploring the associations between other validated indicators of method type and counseling content would be useful to further understand this relationship.

We also note that the MII and MII+ were designed to capture only a small portion of the elements of informed choice. Neither measure, for example, captures outcomes related to counseling bias,which research has shown is of particular salience to LARC methods [37,38]. Further exploration into how to accurately measure other aspects of informed choice beyond content discussed during counseling, as well as other domains of contraceptive autonomy, is an essential next step in understanding quality of care and autonomous decision-making in family planning. New indicators of autonomy should be deployed in large-scale, population-based surveys, so informed, full, and free choice in contraceptive decision-making can be better understood. Other limitations include that women were asked the questions that make up the MII+ about when they last obtained their method. Thus, women who have been using their method for a lengthy period may experience from potential recall bias, which may be especially prominent for LARC users, given the longer duration of these methods. This study was strengthened by the large, nationally representative sample of women included across 6 sub-Saharan African countries, and the use of widely adopted measures of contraceptive counseling content.

We find that regardless of method duration of action, many contraceptive clients are not provided complete information about other methods, method switching, potential side effects, and how to handle side effects during counseling. We also find that women who were using LARC were more likely to have received more complete contraceptive counseling content. Programs and services that aim to meet the reproductive health needs of women seeking contraception should work to ensure basic and essential components of information are delivered to all clients, regardless of which method they plan to adopt. (Table 1)

**Table 1:** Demographic characteristics of women using modern contraceptive methods in six sub-Saharan African countries, 2019–2020

	Burkina Faso	Côte d'Ivoire	Democratic Republic of Congo	Kenya	Nigeria	Uganda
N	1468	720	753	3700	322	1005
Age (years)						
15–24	25%	28%	27%	22%	10%	29%
25–34	39%	40%	43%	44%	38%	42%
35–49	36%	32%	30%	34%	52%	29%
Married	91%	70%	67%	83%	90%	79%
Never attended school	60%	21%	5%	3%	10%	5%
Urban	26%	61%	100%	31%	91%	31%
Parity						
0	5%	11%	12%	3%	6%	5%
1–2	30%	36%	36%	40%	25%	34%
3–4	28%	26%	27%	34%	43%	28%
5+	38%	27%	24%	23%	26%	32%
Current most effective method of contraception						
Implant	53%	29%	44%	42%	33%	36%
IUD	5%	4%	–	4%	10%	6%
Injectable	30%	28%	20%	38%	23%	41%
Pill	11%	31%	10%	8%	18%	6%
Emergency contraception	–	8%	17%	1%	12%	–
Standard days/cycle beads	–	–	4%	1%	–	3%
Female sterilization	–	–	–	5%	–	6%
Received family planning from public facility	89%	60%	45%	75%	57%	62%

“—“ Missing indicates sample size was too small to produce reliable estimates at the population level.

**Table 2:** Aspects of the method information index plus comparing long-acting reversible contraceptive users and other modern method users in six sub-Saharan African countries, 2019–2020

	Burkina Faso		Côte d'Ivoire		The Democratic Republic of Congo		Kenya		Nigeria		Uganda	
	LARC	Non-LARC	LARC	Non-LARC	LARC	Non-LARC	LARC	Non-LARC	LARC	Non-LARC	LARC	Non-LARC
	N = 855	N = 613	N = 235	N = 485	N = 343	N = 410	N = 1732	N = 1968	N = 138	N = 184	N = 419	N = 587
Told about side effects	62%	55%	60%	40%	79%	34%	75%	64%	86%	48%	78%	55%
Told what to do if experienced side effects	58%	45%	49%	31%	71%	24%	70%	56%	79%	42%	72%	44%
Told about methods other than current method	70%	65%	63%	48%	53%	29%	82%	71%	91%	60%	80%	59%
Told they could switch to another method	71%	70%	71%	48%	51%	36%	83%	71%	76%	59%	83%	60%
MII+	46%	40%	32%	21%	36%	8%	59%	45%	59%	36%	60%	35%

**Table 3:** Crude and adjusted^a^ prevalence ratios and 95% confidence intervals for the association between LARC use and the Method Information Index Plus in six sub-Saharan African countries, 2019–2020

Country	Crude prevalence ratio (95% confidence interval)	Adjusted prevalence ratio (95% confidence interval)
Burkina Faso	1.15 (0.92, 1.45)	1.16 (0.91, 1.48)
Côte d'Ivoire	1.55 (1.10, 2.18)	1.13 (0.87, 1.45)
Democratic Republic of Congo	4.32 (1.95, 9.56)	3.65 (1.52, 8.75)
Kenya	1.32 (1.21, 1.43)	1.26 (1.15, 1.38)
Nigeria	1.66 (0.59, 4.68)	1.28 (1.07, 1.54)
Uganda	1.70 (1.48, 1.95)	1.64 (1.42, 1.89)

a Adjusted for age, marital status, parity, urbanicity, facility ownership, and facility type.

## Data availability

The data used in this analysis are publicly available at www.pmadata.org.

## Disclosures and Funding

The authors have no competing interests or conflicts of interest to disclose.

Funding: BB's contribution was supported by a National Research Service Award (T32 HD52468). BB and KT's contribution was supported by an infrastructure grant for population research (P2C HD047879) to the Carolina Population Center at the University of North Carolina at Chapel Hill. LS's contribution was supported by a Ruth L Kirschstein National Research Service Award (T32 HD049302) and Population Research Infrastructure grant (P2C HD047873). The Eunice Kennedy Shriver National Institute of Child Health and Human Development (NICHD) of the National Institutes of Health (NIH) awarded these grants. Contributions by CK, LZ, PZA, FMO, MSZ, SPK, FEM, SS, and members of the PMA PI Group were supported by grants OPP1198333 and OPP1198339 awarded by the Bill and Melinda Gates Foundation. The contents of this article are solely the responsibility of the authors and do not necessarily represent the official views of the NIH/NICHD or the Bill and Melinda Gates Foundation.

The funders of the study had no role in study design, data collection, data analysis, data interpretation, or writing of the report.
